# Project DyAdd: Non-linguistic Theories of Dyslexia Predict Intelligence

**DOI:** 10.3389/fnhum.2020.00316

**Published:** 2020-08-14

**Authors:** Marja Laasonen, Pekka Lahti-Nuuttila, Sami Leppämäki, Pekka Tani, Jan Wikgren, Hanna Harno, Henna Oksanen-Hennah, Emmanuel Pothos, Axel Cleeremans, Matthew W. G. Dye, Denis Cousineau, Laura Hokkanen

**Affiliations:** ^1^Department of Psychology and Logopedics, University of Helsinki, Helsinki, Finland; ^2^Department of Otorhinolaryngology and Phoniatrics – Head and Neck Surgery, Helsinki University Hospital and University of Helsinki, Helsinki, Finland; ^3^Department of Psychiatry, Helsinki University Hospital and University of Helsinki, Helsinki, Finland; ^4^Department of Psychology, Centre for Interdisciplinary Brain Research, University of Jyväskylä, Jyväskylä, Finland; ^5^Clinical Neurosciences, Department of Neurology, Helsinki University Hospital and University of Helsinki, Helsinki, Finland; ^6^Pediatric Neuropsychiatric Unit, Department of Child Psychiatry, Helsinki University Hospital and University of Helsinki, Helsinki, Finland; ^7^Department of Psychology, City University of London, London, United Kingdom; ^8^Center for Research in Cognition & Neurosciences, Université libre de Bruxelles, Brussels, Belgium; ^9^National Technical Institute for the Deaf, Rochester Institute of Technology, Rochester, NY, United States; ^10^School of Psychology, University of Ottawa, Ottawa, ON, Canada

**Keywords:** dyslexia, ADHD, temporal processing, procedural learning, eyeblink conditioning, visual processing, visual attention, comorbidity

## Abstract

Two themes have puzzled the research on developmental and learning disorders for decades. First, some of the risk and protective factors behind developmental challenges are suggested to be shared and some are suggested to be specific for a given condition. Second, language-based learning difficulties like dyslexia are suggested to result from or correlate with non-linguistic aspects of information processing as well. In the current study, we investigated how adults with developmental dyslexia or ADHD as well as healthy controls cluster across various dimensions designed to tap the prominent non-linguistic theories of dyslexia. Participants were 18–55-year-old adults with dyslexia (*n* = 36), ADHD (*n* = 22), and controls (*n* = 35). Non-linguistic theories investigated with experimental designs included temporal processing impairment, abnormal cerebellar functioning, procedural learning difficulties, as well as visual processing and attention deficits. Latent profile analysis (LPA) was used to investigate the emerging groups and patterns of results across these experimental designs. LPA suggested three groups: (1) a large group with average performance in the experimental designs, (2) participants predominantly from the clinical groups but with enhanced conditioning learning, and (3) participants predominantly from the dyslexia group with temporal processing as well as visual processing and attention deficits. Despite the presence of these distinct patterns, participants did not cluster very well based on their original status, nor did the LPA groups differ in their dyslexia or ADHD-related neuropsychological profiles. Remarkably, the LPA groups did differ in their intelligence. These results highlight the continuous and overlapping nature of the observed difficulties and support the multiple deficit model of developmental disorders, which suggests shared risk factors for developmental challenges. It also appears that some of the risk factors suggested by the prominent non-linguistic theories of dyslexia relate to the general level of functioning in tests of intelligence.

## Introduction

Comorbidity between developmental and learning disorders is very common. Accordingly, it has been suggested that various developmental challenges result from risk and protective factors, some of which are shared and some specific for a given condition (Pennington, 2006; Pennington and Bishop, 2009). Related to this, language-based learning difficulties like dyslexia have been suggested to result from or correlate with non-linguistic aspects of information processing. In the current study, we investigate how adults with developmental dyslexia (dyslexia, DD) or attention deficit hyperactivity disorder (ADHD) and controls cluster across various dimensions designed to tap the prominent non-linguistic theories of dyslexia.

Developmental dyslexia is among the most intensively investigated developmental challenges. Despite the amount of research, the causative, correlative, and resulting as well as shared and differentiating factors with other developmental challenges, such as ADHD, are yet to be confirmed. Dyslexia is most often considered to belong to a continuum of language-based developmental and learning difficulties and impaired phonological processing is considered to be its proximal cognitive cause (Wagner, 1986; Torgesen et al., 1994; Snowling, 1995; Boets et al., 2013). Some researchers suggest, however, that impaired phonological processing is only an endophenotype that increases the risk for dyslexia (Snowling and Melby-Lervåg, 2016) or that the phonological processing and reading difficulties that characterize dyslexia could result from a more general cognitive—but non-linguistic—processing impairments.

One of the oldest non-linguistic hypotheses of dyslexia suggests that a general temporal processing impairment results in poorly defined phonological representations and, therefore, in difficulties in grapheme–phoneme mapping and ultimately in poor reading (Tallal, 1980). Another hypothesis suggests that dyslexic readers suffer from abnormal cerebellar functioning, which results in articulatory problems that lead to poor phonological representations and processing as well as to poor general skill and knowledge automatization (Nicolson and Fawcett, 2007, 2011). Related to this, dyslexia has been suggested to be explained by impaired procedural but intact declarative learning (the procedural deficit hypothesis) (Ullman, 2004; Ullman and Pullman, 2015). Finally, difficulties in visual processing and especially attention have been suggested to result in poor reading as well, because reading is a process that stresses the visual system.

Consensus as to whether dyslexia is caused by a purely phonological deficit or if more general, non-linguistic, deficits are involved has not been reached at this point. Proponents of the phonological deficit hypothesis suggest that other difficulties are comorbid or result from the phonological and reading difficulties or from reduced reading experience (Goswami, 2015; Huettig et al., 2018). On the other hand, the more general non-linguistic explanations of dyslexia have been defended based on findings suggesting that (i) the phonological representations in dyslexia might not be impoverished (Ramus and Szenkovits, 2008; Boets et al., 2013), (ii) not all those with dyslexia have phonological difficulties (Valdois et al., 2011), and (iii) some who have phonological difficulties do not have dyslexia (Snowling, 2008; Snowling and Melby-Lervåg, 2016). Thus, phonological skills alone do not fully explain variation in reading abilities (Kibby et al., 2014). Likewise, no single cognitive factor alone can explain all the behavioral variation in every individual with dyslexia (Ramus and Ahissar, 2012). All this suggests that characteristics of developmental disorders are multiple, continuous, and possibly shared with other developmental challenges.

To resolve some of these open questions, Project DyAdd^1^ tested the prominent non-linguistic theories of dyslexia, at different levels of analysis, in adults with developmental dyslexia or ADHD as well as in healthy controls with the main objective of defining the differentiating and shared characteristics. Neurocognitive difficulties were investigated with clinical neuropsychological methods (behavioral level) (Laasonen et al., 2009c, 2010; Kivisaari et al., 2012), and basic cognitive functions were assessed with experimental methods (cognitive level). Biological measures used in the project were serum lipid fatty acids and measures of cerebellar functioning (biological level). Abnormalities in fatty acid metabolism have been suggested to contribute to both ADHD and dyslexia as well as their cognitive and behavioral profiles (as reviewed by Laasonen et al., 2009a, b). Similarly, the cerebellum has been implicated to contribute to the behavioral and cognitive profile of dyslexia (Nicolson et al., 2001). Associations between neuropsychological, experimental, and biological measures were studied as well (Laasonen et al., 2009a, b). The experimental paradigms of Project DyAdd targeted the prominent non-linguistic theories of developmental dyslexia, that is, temporal processing impairment, abnormal cerebellar functioning, procedural learning difficulties, as well as visual processing and attention deficits.

Below, we shortly describe our previous results for the four paradigms used in the current study. These include group differences between healthy controls, adults with developmental dyslexia or ADHD, as well as correlations between the performance in the experimental paradigms and dyslexia-related and ADHD-related cognition.

Temporal processing was assessed with tasks where the participant judged the order or the simultaneity/non-simultaneity of visual stimuli (Sarkio, 2009). The group differences have not been published, but in our other studies with similar tasks, impaired temporal processing has been found in adults with dyslexia across sensory modalities and their combinations (Laasonen et al., 2001, 2002a,b; Virsu et al., 2003). Further, in our previous studies, temporal processing has been shown to correlate with phonological processing in both dyslexic and fluent readers (Laasonen et al., 2001, 2002b, 2012c; Laasonen, 2002; Virsu et al., 2003). Taken together, we have shown that temporal processing impairment associates with dyslexia and dyslexia-related cognition of phonological processing.

We investigated the role of the cerebellum with two paradigms of classical eye-blink conditioning (Laasonen et al., 2012a). The group with dyslexia was slower overall in their learning compared to the control group and had pronounced difficulties in a medio-temporal-dependent paradigm compared to the more cerebellum-dependent paradigm. Over all groups, responses in the cerebellum-dependent paradigm correlated positively with reading performance and, within those who acquired conditioned behavior, responses of the medio-temporal-dependent paradigm correlated positively with spelling. Taken together, we showed that cerebellum-based classical eye-blink conditioning did not associate with dyslexia, although it did relate to dyslexia-related cognition of reading.

Procedural learning was investigated by us with two paradigms (Laasonen et al., 2014). The groups with dyslexia and ADHD did not differ from each other or controls in sequence learning, but only the control group learned the grammar in an artificial grammar learning (AGL) task. Total group correlations indicated that explicit knowledge of the grammar correlated positively with phonological processing and reading performance. No correlations were found for the implicit knowledge. Taken together, in our previous study, impaired procedural learning was associated with both dyslexia and ADHD but only with dyslexia-related cognition, that is, phonological processing and reading.

We investigated visual attention processes with three paradigms (Laasonen et al., 2012b). Adults with dyslexia were not impaired in their capacity of visual attention but had difficulties in temporal and spatial aspects. The ADHD group did not have any difficulties in the tasks. When all the participants were analyzed together, spatial and capacity of visual attention positively predicted performance in phonological processing and reading. Taken together, we showed that visual attention was associated with dyslexia and dyslexia-related cognition, that is, phonological processing and reading.

**FIGURE 1:**
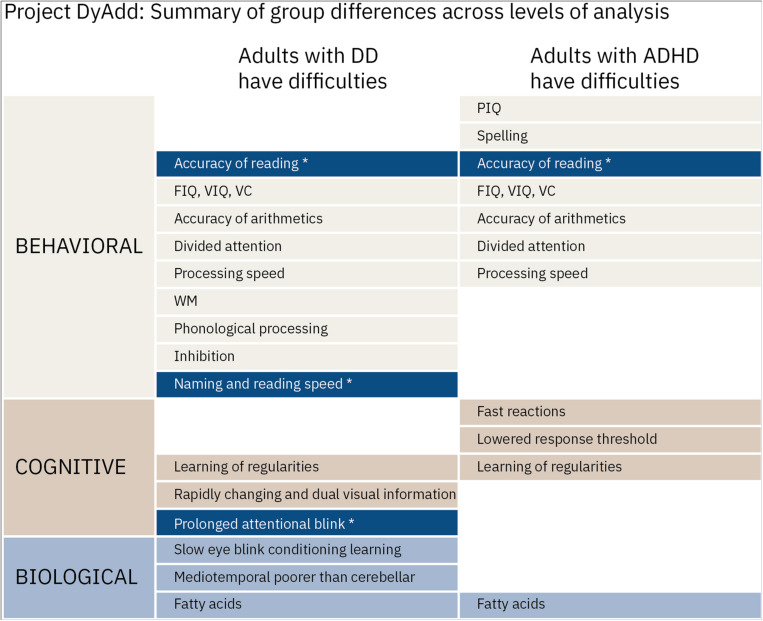
Summary of the published results of project DyAdd. Named difficulties indicate significant differences compared to controls. Asterisks indicate differences where those with dyslexia differed not only from controls but also from those with ADHD. FIQ, full Intelligence quotient; PIQ, performance intelligence quotient; VIQ, verbal intelligence quotient; VC, verbal comprehension; and WM, working memory from the Wechsler Adult Intelligence Scale (Wechsler, 2005). NB, nota bene. Temporal processing is not included in the figure. References to the original articles Laasonen et al., 2009a,b,c,^2010^, 2012a,b, 2001).

In Figure 1, we present a summary of the published results of Project DyAdd across the behavioral, cognitive, and biological levels of analysis. Results presented in Figure 1 and those detailed above indicate that performance in tasks tapping the prominent non-linguistic theories of developmental dyslexia correlates with dyslexia-related cognition when inspected over all participants, that is, phonological processing and reading. However, those with dyslexia are not always impaired in these same tasks compared to controls and it is difficult to differentiate individuals with dyslexia from those with ADHD. All this suggests that the characteristics related to dyslexia are continuous in a way that the associations emerge also in other populations and that the risk factors across developmental difficulties are shared in a way that makes them difficult to differentiate from each other. One possible explanation for the findings is the Pennington’s multiple deficit model (Pennington, 2006; Pennington and Bishop, 2009), which suggests that the continuous nature of a given developmental disorder cannot be explained by a single gene or cognitive factor. Instead, developmental disorders share many probabilistic genetic and environmental risk and protective factors, and this leads to the high comorbidity between them both at the neural, cognitive, and behavioral levels.

In the current study, we re-analyzed the data from Project DyAdd with latent profile analysis (LPA) using measures from the experimental designs probing the prominent non-linguistic theories of dyslexia, that is, temporal processing impairment, abnormal cerebellar functioning, procedural learning difficulties, and visual attention deficits. We investigate how adults with developmental dyslexia or ADHD and a healthy control group cluster when all the experimental designs are considered at the same time and whether specific profiles of difficulties can be identified. The profiles of the groups emerging from LPA are investigated further across domains of neuropsychological functioning that characterize dyslexia and ADHD as well as general level of functioning in tests of intelligence. We hypothesize that dyslexia and ADHD will not emerge as separate groups in the LPA with the possible exception of time-constrained sequential processing (see the summary of Figure 1). The neuropsychological profiles of the LPA groups are expected to reflect this as well. Consequently, we expect not to find dyslexia-specific or ADHD-specific profiles in the LPA groups.

## Materials and Methods

Description of the general methods of project DyAdd can be found in a previous article (Laasonen et al., 2009c).

### Participants

Participants in the current study were those who participated in project DyAdd and its experimental tasks (Laasonen et al., 2012a, b, 2014). General inclusion criteria were as follows: Finnish as the native language, age between 18 and 55 years, and Wechsler Abbreviated Scale of Intelligence–Full intelligence quotient, WASI FIQ (Wechsler, 1999, 2005), over 70 because of the ICD-10 criteria for specific reading disorder (World Health Organization, 1998). General exclusion criteria were brain injury, somatic or psychiatric condition affecting cognitive functions (including major depression), psychotropic drugs affecting cognitive functions, and substance abuse. Blood samples were collected to rule out endocrinopathies (e.g., dysfunction of the thyroid gland), diabetes, renal dysfunction, abuse of alcohol, and similar somatic states that might compromise cognitive functions. Laboratory tests included hemoglobin, red blood count, white blood count, platelet count, thyroid stimulating hormone, serum creatinine, alanine aminotransferase, gamma-glutamyltransferase, and fasting blood glucose.

Participants in the dyslexia group (*n* = 36) had a history of reading difficulties and a prior diagnosis. Their phonological processing and reading performance were assessed at the time of the study. All performed 1 SD below the mean in both, with the exception of one participant with poor residual phonological processing only (Laasonen et al., 2009c) as assessed with phonological naming [rapid alternate stimulus naming (RAS) speed/accuracy, Wolf, 1986], phonological awareness (phonological synthesis accuracy, Laasonen et al., 2002b), phonological memory (WAIS digit span forward length, Wechsler, 2005), and reading (oral reading speed/accuracy, task details in Laasonen et al., 2002b). ADHD diagnosis and a history of ADHD-related difficulties were exclusion criteria. The latter was screened with the Wender Utah Rating Scale (WURS) (Ward et al., 1993) and the Adult Problem Questionnaire (APQ) (De Quiros and Kinsbourne, 2001).

Participants with ADHD (*n* = 22) had a history of ADHD-related difficulties and a prior diagnosis based on the DSM-IV criteria (American Psychiatric Association, 1994) using CAADID (Epstein et al., 2001) by a medical doctor specialized in neuropsychiatry (author SL or PT in most cases). Participants with any of the three subtypes of ADHD were eligible for the study. Confounding psychiatric disorders were excluded by structured diagnostic interviews (SCID-I and SCID-II) (First et al., 1996, 1997). Dyslexia diagnosis and a history of dyslexia-related difficulties were exclusion criteria. The latter was screened with Adult Reading History Questionnaire (ARHQ) (Lefly and Pennington, 2000). Participants with ADHD participated in the project unmedicated. A wash-out period of at least 1 week was required before and during the study appointments if they were using methylphenidate. Those with medication with a longer half-life were excluded from the project. Exclusion criteria for the Control group (*n* = 35) were a history of reading or ADHD-related difficulties or a prior diagnosis of dyslexia or ADHD.

### Experimental Designs

Detailed description of the experimental tasks and procedures can be found in previous articles (Laasonen et al., 2012a, b, 2014). Below, we present the variables used and, in case of composites, their Cronbach’s alpha reliabilities.

Temporal processing (Sarkio, 2009) was assessed with two visual tasks, which were both realized with gray or green stimuli on a black background. (1) Temporal order judgment (TOJ) assessed participant’s 74% correct threshold in milliseconds in assessing the order of two visual stimuli that were presented one above the other. (2) Temporal processing acuity (TPA) estimated the 74% correct threshold for assessing correctly the simultaneity/non-simultaneity of streams of three visual stimuli, which were presented one stream above the other. For this study, we collapsed four variables (thresholds: gray or green × TPA or TOJ) into a single measure using a principal component analysis (PCA) over all the groups (α = 0.55, removing the threshold for green TPA resulted in α = 0.7). To that end, we calculated and saved the regression-based component scores.

Cerebellar functions were assessed with two classical eye-blink conditioning tasks (Laasonen et al., 2012a). Both included a preconditioning phase (20 trials: randomly presented 10 tones and 10 air puffs to the corner of the eye), a conditioning phase (80 trials: blocks of tones and tones + air puffs), and an extinction phase (20 trials: tones only). Eye-blink responses were recorded with EMG. (1) In the delay conditioning paradigm (DCP), the 800-ms tone and the 100-ms air puff ended simultaneously in the conditioning phase. (2) In the trace conditioning paradigm (TCP), the 100-ms tone and the 100-ms air puff were separated by an interval of 600 ms. The DCP assesses mostly cerebellum-based procedural learning, while the TCP measures mostly declarative learning involving also the medio-temporal areas. Outcome measures were the number of conditioned responses as well as their peak amplitude, peak latency, and magnitude. For this study, we kept two variables: number of conditioned responses in the DCP and in the TCP.

Procedural learning (Laasonen et al., 2014) was assessed with two tasks. (1) The serial reaction time (SRT) task was a choice reaction time task in which the participants did not know that the presentation order of stimuli was defined by a grammar (Knowlton et al., 1992). Stimuli were geometrical non-linguistic shapes, each presented at a constant spatial location, that were presented in blocks (block 1: random, 2–11: structured, 12: random, 13: structured). Learning was expected to result into faster reaction time in the structured compared to random blocks. The outcome measures were the average percentage of erroneous answers and the average reaction time for correct answers per block. Implicit procedural learning was operationalized by comparing the performance in the last random block to the average of the adjacent structured blocks. (2) AGL was assessed with a task where the participants had to memorize horizontal strings of 2–6 geometrical non-linguistic shapes. Afterwards, they were told that the strings followed a set of rules (Abrams and Reber, 1988; Knowlton and Squire, 1996) and classified a new set of strings into grammatical and non-grammatical. The outcome measures were the percentage of correct grammatical and similar answers. The latter was defined by chunk strength, which is based on fragment overlap. Implicit procedural learning was operationalized as better than chance performance in grammatical accuracy. For this study, we used the following four variables. For SRT, we kept accuracy in the last random block divided by average accuracy in adjacent blocks and reaction time in the last random block divided by average reaction time in adjacent blocks; for AGL, we kept grammatical accuracy and similarity ratings.

Visual processing and attention (Laasonen et al., 2012b) were assessed with three tasks. (1) Spatial characteristics of visual attention were estimated with useful field of view (UFOV) where the participant fixated centrally and conducted a yes/no decision to detect the presence or absence of a target (control condition). Some trials required locating an additional peripheral target without distractors (experimental condition without distractors) or with them (experimental condition with distractors). The four outcome measures for each condition were the presentation duration of the stimuli to reach a 79.3% correct threshold for both the central and peripheral task with and without distractors. (2) Temporal characteristics of visual attention were estimated with the attentional blink (AB) paradigm using a similar method to Green and Bavelier (2003). Again, the participant fixated centrally and was presented with black letters (presentation time 26.7 ms with 106.7 ISI), a white letter, the first target to be identified (T1), other black letters, and a black X to be detected, the second target (T2), that appeared in 50% of the trials. A trial consisted of 16–24 letters. Outcome measures were the proportion of correct detection of T2 (baseline), the proportion of correct identification of T1 while correctly detecting T2 (dual task), and, finally, T2 detection accuracy as a function of T1–T2 lag when T1 was correctly identified (dual task), which were used to estimate the four parameters of Cousineau and colleagues (Cousineau et al., 2006): lag-1 sparing, width, amplitude, and minimum. (3) Capacity of visual attention was estimated with multiple object tracking (MOT), where the participant fixated centrally and tracked peripherally 16 randomly moving dots. One, three, five, or seven of the tracked dots were blue and the rest were yellow. After 2 s of movement, all the dots turned yellow and moved for another 5 s. After this, movement stopped, and one of the dots turned white, and the participant made a yes/no decision whether the white dot had been one of the blue targets. The outcome measures were the percent correct as a function of the number of dots to be tracked. For this study, we aggregated the four UFOV variables (thresholds for the four conditions: distractors or no distractors × peripheral stimulus at 7° or 21°) into a single variable by inserting them into a PCA over all the groups (α = 0.6). We also kept for the temporal characteristics two variables: Cousineau parameters for AB length (width) and depth (minimum). Lastly, one variable for capacity was kept: Percent correct for the four MOT conditions (1, 3, 5, or 7 dots to follow) were inserted into a PCA over all the groups in order to get one measure for the four conditions (α = 0.8).

### Domains of Neuropsychological and General Level of Functioning

These tests were included into the neuropsychological assessment battery that was divided into two separate sessions. Detailed description of the neuropsychological tasks can be found in previous articles (Laasonen et al., 2012b). For this study, we used the neuropsychological domains of phonological processing (average of awareness, memory, and naming speed), technical reading (average of speed and accuracy), reading comprehension (average of speed and accuracy), spelling (accuracy), arithmetic (accuracy), executive functions (average of set shifting, inhibition, and planning), and attention (average of sustained and divided). These are presented in more detail in Supplementary Appendix 1. Cronbach’s alpha reliabilities conducted over the variables were acceptable, except for the domain of executive functions. Removing variables from this composite did not enhance its internal consistency.

To assess general level of functioning, we used intelligence, more specifically, four indices from the Wechsler Intelligence Scale for Adults, third revision (Wechsler, 2005). These were verbal comprehension (subtests: similarities, vocabulary), working memory (subtests: arithmetic, digit span, letter–number sequencing), perceptual organization (subtests: block design, matrix reasoning), and processing speed (subtest: digit-symbol coding).

### Statistical Analyses

The variables of the experimental designs are described above. To remove the effect of extreme values in the data, we used 90% winsorizing over all the groups and then substituted the remaining extreme values with the value of the poorest non-outlier. After this, the few missing values were imputed using expectation maximization (EM) techniques over all experimental design variables and participants with the group as two dummy variables. Finally, the variables were *z*-standardized based on the control group values and, when needed, inverted to indicate better performance with positive values resulting in variables with the mean of 0 and SD of 1.

The variables of the neuropsychological domains and general level of functioning are described above. The same neuropsychological composite variables were used as in the previous studies; that is, the scores of all participants were transformed based on the age-corrected performance of the control group and converted, if necessary, to indicate better performance with a larger positive value resulting in variables with a mean of 10 and an SD of 3 (Laasonen et al., 2012b). Regarding intelligence, the standardized norms that are based on the age-corrected performance of the normative group were used and the scores were converted to the same scale as the neuropsychological domains, that is, their mean was also 10 and SD was 3. After this, the few missing values were imputed using EM techniques over all neuropsychological and intelligence composites and participants with the group as two dummy variables. Finally, the neuropsychological composites were restricted to the same scale as the intelligence composites (1–19).

For statistical analyses, LPA was used in order to investigate how the original groups clustered based on the variables retrieved from the experimental designs. Differences in the distribution of participants into the LPA groups as well as differences in the background variables between the LPA groups were analyzed with Chi-squared tests and ANOVAs. The LPA group profiles in the experimental designs as well as domains of neuropsychological and general level of functioning were analyzed with multivariate ANCOVA (a Wilks test) and, in the case of a significant main effect, with one-way ANCOVAs. Level of significance was set at *p* = 0.05 with Bonferroni correction for the *post hoc* tests. More detailed description can be found in the results.

For the literature search presented in the discussion, we searched the Web of Science on December 10, 2019 with the following syntax: TOPIC:(dyslexia) AND ALL FIELDS:(temporal OR implicit OR procedural OR cerebellum OR cerebellar OR vision OR visual). Timespan: Last 5 years. Indexes: SCI-EXPANDED, SSCI, A&HCI, CPCI-S, CPCI-SSH, BKCI-S, BKCI-SSH, ESCI, CCR-EXPANDED, IC.

## Results

### Latent Profile Analysis

Latent profile analysis was used in order to investigate how the original groups (dyslexia, ADHD, control) clustered based on the 11 variables retrieved from the experimental designs. R version 3.2.1 (R Core Team, 2018) with mclust version 5.2 (Scrucca et al., 2016) was used for the analyses. In a nutshell, LPA tries to fit a certain number of multivariate normal distributions on the data so as to maximize the fit. The number of distributions is varied (from 1 to 9); there are also various constraints that are tested (e.g., equal variance, absence of covariance, etc.). The most successful yet parsimonious model, as assessed by a BIC index of fit, is retained. The solution found was a mixture of three distributions (each having zero covariance but distinct variances; e.g., a VII solution; see Scrucca et al., 2016). Loglikelihood was −1462.55 for 55 free parameters.

The three LPA groups (see Table 1) differed greatly in their size, and the distribution of participants in the LPA groups did not mirror very well the participant’s original group [χ^2^(4) = 8.25, *p* = 0.083]. Analyses on the background variables indicated that gender, handedness, and level of education did not differentiate the LPA groups, but age did (see Table 1). Bonferroni-corrected *post hoc* tests showed that those in the LPA3 were older than those in LPA1 (*p* = 0.037) or LPA2 (*p* < 0.001). Thus, age was used as a covariate in the following analyses.

**TABLE 1:** Number of participants in the original and LPA groups as well as background variables.

		**LPA-generated groups**			**Total F/x^2^**
		**LPA1**	**LPA2**	**LPA3**	

**Original groups**					
Dyslexia		19 (53%)	7 (19%)	10 (28%)	36
ADHD		16 (73%)	5 (23%)	1 (5%)	22
Control		27 (77%)	4 (11%)	4 (11%)	35
Total		62 (67%)	16 (17%)	15 (16%)	93
Age in years	Mean	35.60	29.25	42.80	(2,90) = 7.40**
	SD	(10.43)	(8.23)	(8.46)	
Gender					
Female	Count	29	7	8	(2) = 0.31
Male	Count	33	9	7	
Handedness					
Right	Count	54	16	13	(2) = 2.33
Left	Count	8	0	2	
Ambi	Count	0	0	0	
Education^1^					
Basic	Count	28	9	6	(4) = 2.87
Middle	Count	14	4	6	
High	Count	19	3	3	

**p < 0.01. ^1^Basic, primary and secondary education; middle, vocational high school; high, university.

### LPA Group Profiles in Experimental Designs

**FIGURE 2:**
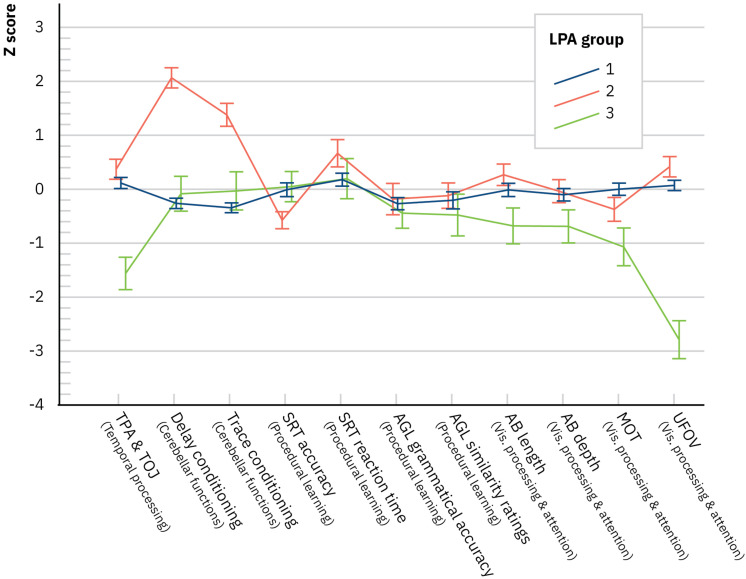
Latent profile analysis groups’ performance in the experimental designs (mean with SE). AB, attentional blink; AGL, artificial grammar learning; MOT, multiple object tracking; SRT, serial reaction time; TPA, temporal processing acuity; TOJ, temporal order judgment; UFOV, useful field of view. NB, nota bene. Presented values are not corrected for the covariate age.

The profiles of the LPA groups were inspected with a multivariate ANCOVA (a Wilks test) where the LPA group was the between-subjects factor and the variables of the experimental designs were the multivariate factors (in *z*-scores) of the dependent measure and age as the covariate. The difference between the LPA groups was significant, *F*(22,158) = 11.37, *p* < 0.001, Λ = 0.15, and η${}_{\text{p}}^{2}$ = 0.61. This result indicates that the LPA groups differed strongly in their overall pattern of performance in the experimental designs. Using the temporal processing composite with better internal consistency did not affect the results [*F*(22,158) = 10.75, *p* < 0.001, Λ = 0.16, and η${}_{\text{p}}^{2}$ = 0.60]. In follow-up ANCOVAs for the experimental designs, significant differences between the LPA groups emerged in temporal processing [*F*(2,89) = 19.63, *p* < 0.001, η${}_{\text{p}}^{2}$ = 0.31] where those in the LPA3 group were slower compared to the other groups (Bonferroni-corrected comparisons for estimated marginal means, all *p*s < 0.001), cerebellar functions [delay conditioning, *F*(2,89) = 43.65, *p* < 0.001, η${}_{\text{p}}^{2}$ = 0.50, with LPA2 having more conditioned responses than the other groups (all *p*s < 0.001)], trace conditioning [*F*(2,89) = 23.47, *p* < 0.001, η${}_{\text{p}}^{2}$ = 0.35, with LPA2 having again more conditioned responses than the other groups (all *p*s < 0.001)], procedural learning [SRT accuracy, *F*(2,89) = 3.39, *p* = 0.038, η${}_{\text{p}}^{2}$ = 0.07, with the Bonferroni corrections, comparisons for estimated marginal means were not significant], and visual processing and attention [UFOV, *F*(2,89) = 58.55, *p* < 0.001, η${}_{\text{p}}^{2}$ = 0.57, with LPA3 being poorer than the other groups (all *p*s < 0.001); MOT, *F*(2,89) = 6.48, *p* = 0.002, η${}_{\text{p}}^{2}$ = 0.13, with LPA3 poorer than LPA1 (*p* = 0.003)]. Figure 2 depicts the LPA group’s mean performance in the experimental designs. LPA1 performed on average within −1 to +1 SD in all assessed areas. LPA2 performed on average within −1 to +1 SD in all areas, except for the number of conditioned responses that were large. LPA3 was poor in visual processing and attention as well as temporal processing.

### LPA Group Profiles in Domains of Neuropsychological and General Level of Functioning

**FIGURE 3:**
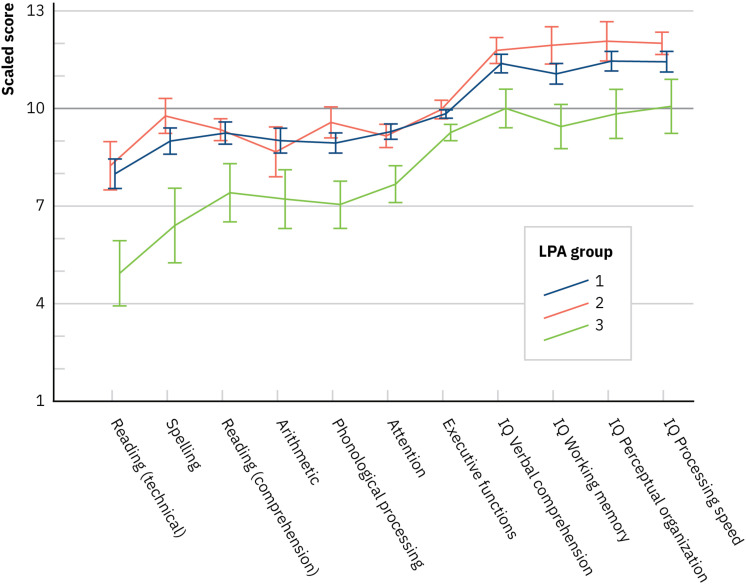
Latent profile analysis groups’ performance in the domains of neuropsychological and general level of functioning (mean with SE). IQ, intelligence quotient (Wechsler, 2005). NB, nota bene. Presented values are not corrected for the covariate age.

Next, we inspected the profiles of the LPA groups across the neuropsychological domains, again, with a multivariate ANCOVA (a Wilks test) where the LPA group was the between-subjects factor and the neuropsychological domains as the multivariate factors (in standardized scores) of the dependent measure and age as the covariate. The main effect of LPA group was not significant, *F*(14,166) = 1.288, *p* = 0.219, Λ = 0.81, and η${}_{\text{p}}^{2}$ = 0.098, indicating that the groups did not differ in their dyslexia- or ADHD-related neuropsychological performance. Removing the executive functioning composite with poor internal consistency from the analysis did not affect the results [main effect of LPA group, *F*(12,168) = 1.44, *p* = 0.153, Λ = 0.822, and η${}_{\text{p}}^{2}$ = 0.093]. Further, a multivariate ANCOVA (a Wilks test) over the separate executive function variables of the composite, described in Supplementary Appendix 1, resulted in a non-significant main effect of LPA group as well [*F*(14,160) = 1.46, *p* = 0.130, Λ = 0.786, and η${}_{\text{p}}^{2}$ = 0.114]. Figure 3 depicts the LPA groups’ performance in the domains neuropsychological functioning (the seven points to the left of the plot). All the LPA groups performed on average within −1 to +1 SD in all assessed areas, except in technical reading and spelling.

For the measures of intelligence in standardized scores, the results appeared somewhat different. Now, the main effect of the LPA group was significant [*F*(8,172) = 2.086, *p* = 0.040, Λ = 0.83, η${}_{\text{p}}^{2}$ = 0.09]. One-way ANCOVAs with age as a covariate indicated that the LPA groups differed in all the subdomains, that is, verbal comprehension [*F*(2,89) = 3.22, *p* = 0.045, η${}_{\text{p}}^{2}$ = 0.07], working memory [*F*(2,89) = 4.74, *p* = 0.011, η${}_{\text{p}}^{2}$ = 0.10], perceptual organization [*F*(2,89) = 4.96, *p* = 0.009, η${}_{\text{p}}^{2}$ = 0.10], and processing speed [*F*(2,89) = 5.39, *p* = 0.006, η${}_{\text{p}}^{2}$ = 0.11]. Bonferroni-corrected comparisons for estimated marginal means indicated that LPA3 was poorer than the other groups in working memory (*p*s < 0.045), perceptual organization (*p*s < 0.026), and processing speed (*p*s < 0.026), and almost in verbal comprehension (*p*s < 0.073). Figure 3 depicts the LPA groups’ performance in the general level of functioning (the four last points to the right of the plot). LPA1 and LPA2 performed on average within 0 to +1 SD in all assessed areas, whereas those in the LPA3 performed at −1 to 0.

## Discussion

In the current study, we investigated how adults with developmental dyslexia, ADHD, and controls cluster across various dimensions designed to tap the prominent non-linguistic theories of dyslexia. Tested domains included temporal processing impairment, abnormal cerebellar functioning, procedural learning difficulties, and visual attention deficits. LPA was conducted over all participants and experimental designs.

First, we hypothesized that dyslexia and ADHD would not emerge as separate groups in the LPA with the possible exception of time-constrained sequential processing (see the summary of results in Figure 1). The results showed indeed that the participants did not group very well based on their original status. Instead, the LPA resulted in three groups: the largest LPA1 group with 67% of the participants had average performance in the experimental designs. This indicates that most participants do not have difficulties in any of the experimental tasks whether they belong to the group of controls, ADHD, or dyslexia. The second LPA2 group with 17% of the participants consisted of participants predominantly from the clinical groups who exhibited enhanced conditioning learning. Age is one of the factors that is well known to have an effect on conditioning learning (Woodruff-Pak, 2002) and of the background variables, participants in the LPA2 group were the youngest. However, as participant age was controlled in the analyses, age or factors closely related to it cannot explain the finding of enhanced conditioning. There are multiple other factors that might have been unevenly distributed across our LPA groups but were not, unfortunately, assessed. For example, anxiety and the temperamental trait of behavioral inhibition covary with enhanced conditioning learning (Caulfield et al., 2013; Allen et al., 2019). The third LPA3 group with 16% of the participants, predominantly from the dyslexia group, had difficulties in temporal processing as well as in visual processing and attention, a finding in line with our expectations related to time-constrained sequential processing. Also, these types of tasks are known to be affected by increasing age (Laasonen et al., 2002a; Virsu et al., 2003), and this group was the oldest one. However, as noted above, age was used as a covariate in all the analyses.

Second, we expected that the neuropsychological profiles of the LPA groups would reflect the fact that dyslexia, ADHD, and healthy controls could not be separated in a way that we would find dyslexia-specific or ADHD-specific profiles in the LPA groups. The results confirmed this, as the LPA groups did not differ in their dyslexia or ADHD-related neuropsychological profiles.

These two sets of results together align with the suggestions of Pennington’s multiple deficit model (Pennington, 2006; Pennington and Bishop, 2009) as it appears that the original groups of the current study share many risk and perhaps also protective factors, which lead to overlapping LPA groups and to the high similarity between LPA groups at the neuropsychological level. Inherent to the multiple deficit model is that the risk and protective factors are continuous. In line with this, we have shown that those with developmental dyslexia are poorer in temporal processing compared to fluent readers but in a way that the distribution of their performance is restricted to the areas of poor and mostly average performance, none of them reaching the threshold of above average performance (Service and Laasonen, 2019). Thus, the place of the distribution for risk and protective factors might vary across conditions and with sampling, sometimes resulting in significant group differences.

The most remarkable finding of the current study was that the LPA groups that were formed based on their performance in tasks designed to tap the non-linguistic theories of dyslexia differed most clearly in their intelligence. The third LPA3 group with difficulties in temporal processing as well as visual processing and attention exhibited lower scores than the other groups across the standardized and age-corrected IQ indices, that is, working memory, perceptual organization, processing speed, and at a trend level in verbal comprehension. This pattern of results indicates differences in the levels of severity across the different LPA groups and suggests that the group with the lowest IQ score, although at average, also had difficulties in temporal processing and in visual processing and attention. This finding did not generalize to abnormal cerebellar functioning or procedural learning difficulties.

Inspired by this finding, we searched for original research and review articles (as well as articles cited by these reviews) published during the last 5 years on the topic of dyslexia and temporal processing, cerebellar functions, procedural learning, or visual processing and attention. Surprisingly, a pattern emerged again. For publications on temporal processing and visual processing and attention, only very seldom were the group IQs reported or compared in a way that group-level matching requires. Most often, the groups were characterized as having normal IQ or the exact values were not reported. For example, for temporal or magnocellular processing, papers presented either no or insufficient information on IQ, or group-level matching was imperfect (Gori et al., 2016; Moll et al., 2016; Casini et al., 2018; Fostick and Revah, 2018; Mascheretti et al., 2018; Stefanac et al., 2019). For visual attention or processing, IQs were not reported or matched between the groups (Bosse and Valdois, 2003; Bosse et al., 2007; Germano et al., 2014; Lobier and Valdois, 2015; Zoubrinetzky et al., 2016). Thus, conducting a meta-analysis on the subject became impossible. This was reflected in our results, where IQ appeared to covary with especially temporal processing as well as visual processing and attention. Also in our previous studies, performance in tasks of temporal processing (Laasonen et al., 2001; Laasonen, 2002) as well as visual processing and attention (unpublished analyses from Laasonen et al., 2012b) has correlated with measures of intelligence. One has to ask, then, whether some of the non-linguistic theories of dyslexia predict also minor variations in intelligence. Historically, a discrepancy between poorer reading and better intelligence was required for the identification of a specific reading disability (Rutter and Yule, 1975). Later, the importance of IQ has been emphasized less (Morris and Fletcher, 1988). In the future, although strict IQ or IQ-reading discrepancy criteria for dyslexia might not be justifiable, research focusing on non-linguistic correlates of dyslexia should consider the role of other possibly explaining factors for their findings more rigorously, including age and especially intelligence.

One intriguing possibility that could explain the current findings is that intelligence and reading or its difficulties do covary to some extent after all. Recent results in the area of genetics provide support for this. For example, a general genetic factor has been suggested that would explain variation in both non-verbal intelligence and reading (Lazaroo et al., 2019), and significant overlap between word reading and intelligence has emerged in a recent genome-wide association study (Price et al., 2020). Further, it has been shown for dyslexia that there is an interrelation between genotype, brain anatomy, and neurofunctionality (Skeide et al., 2015, 2016; Neef et al., 2017). All this points to a multifactorial and multigenetic background for dyslexia that has a role for both intelligence and perhaps also non-linguistic processing.

In our statistical analyses, the clinical groups did not cluster into corresponding LPA groups, nor did the LPA groups differ in their neuropsychological functioning although intelligence differentiated between them. However, Table 1 suggests that there were rather many dyslexic readers in the LPA3 group. Further, Figure 3 suggests that the LPA groups could be interpreted to reflect levels of severity across tasks of dyslexia-related and ADHD-related cognition, in addition to intelligence. Specifically, it appears that the LPA3 group with many dyslexic readers had difficulties in temporal processing as well as in visual processing and attention, that is, in time-constrained sequential processing. LPA3 was the most impaired also across the areas of neuropsychological functioning and intelligence, although, in our analyses, the differences did not always reach statistical significance. In the future, focusing on both the non-linguistic aspects of performance as well as intelligence with larger sample sizes may increase our understanding of the condition and possibly form a fruitful basis for prediction and early diagnosis (Mannel et al., 2015; Muller et al., 2016). Our current sample size might not have been large enough to reveal all the significant effects, and a preplanned sample size could have led to more adequate power (Tabachnick and Fidell, 2014).

## Conclusion

In the current study, we investigated how adults with developmental dyslexia or ADHD and controls cluster across various dimensions designed to tap the prominent non-linguistic theories of dyslexia. Tested domains included temporal processing impairment, abnormal cerebellar functioning, procedural learning difficulties, and visual attention deficits. Our results highlight the continuous and overlapping nature of the observed difficulties and support the multiple deficit model of developmental disorders, which suggests shared risk factors for developmental challenges. Further, it appears that some of the risk factors suggested by the prominent non-linguistic theories of dyslexia are related to the general level of functioning in tests of intelligence.

## Data Availability Statement

The data analyzed in this study is subject to the following licenses/restrictions: Datasets are available on request. Requests to access these datasets should be directed to marja.laasonen@helsinki.fi.

## Ethics Statement

The studies involving human participants were reviewed and approved by Ethical Board of the Helsinki Uusimaa Hospital District. The patients/participants provided their written informed consent to participate in this study.

## Author Contributions

ML is the PI of Project DyAdd and responsible for the original idea, statistical analyses, and writing of the article. PL-N is responsible for the statistical design as well as the illustrations of the article. SL and PT are responsible for the ADHD and HH for the cerebellar patient expertise and diagnoses in the Project DyAdd. JW is responsible for the eyeblink designs, and EP and AC are responsible for the procedural learning designs in the Project DyAdd. HO-H participated in the latter as a postgraduate researcher and contributed to an original publication. MD and DC are responsible for the visual attention designs and DC for their analysis in the original publication of the Project DyAdd. LH is responsible for the clinical neuropsychological expertise on ADHD and participated as the second core senior to the project DyAdd in addition to ML. All authors have contributed substantially to the conception or design of the work or the acquisition, analysis, or interpretation of data for the work. All authors have collaborated on drafting the work and revising it critically for important intellectual content. All authors have given their final approval of the version to be published.

## Conflict of Interest

The authors declare that the research was conducted in the absence of any commercial or financial relationships that could be construed as a potential conflict of interest.

## Footnotes

1 https://www.helsinki.fi/en/researchgroups/project-dyadd

## References

[B1] Abrams M. Reber A. S. (1988). Implicit learning: robustness in the face of psychiatric disorders. *J. Psycholinguist. Res.* 17 425–439. 10.1007/bf01067228 3193378

[B2] Allen M. T. Myers C. E. Beck K. D. Pang K. C. H. Servatius R. J. (2019). Inhibited Personality temperaments translated through enhanced avoidance and associative learning increase vulnerability for PTSD. *Front. Psychol.* 10:496. 10.3389/fpsyg.2019.00496 30967806 PMC6440249

[B3] American Psychiatric Association (1994). *Diagnostic and Statistical Manual of Mental Disorders.* Washington, DC: APA Press.

[B4] Boets B. Op de Beeck H. P. Vandermosten M. Scott S. K. Gillebert C. R. Mantini D. (2013). Intact but less accessible phonetic representations in adults with dyslexia. *Science* 342 1251–1254. 10.1126/science.1244333 24311693 PMC3932003

[B5] Bosse M. L. Tainturier M. J. Valdois S. (2007). Developmental dyslexia: the visual attention span deficit hypothesis. *Cognition* 104 198–230. 10.1016/j.cognition.2006.05.009 16859667

[B6] Bosse M. L. Valdois S. (2003). Patterns of developmental dyslexia according to a multi-trace memory model of reading. *Curr. Psychol. Lett. Behav. Brain Cogn.* 1 Available online at: https://journals.openedition.org/cpl/92

[B7] Casini L. Pech-Georgel C. Ziegler J. C. (2018). It’s about time: revisiting temporal processing deficits in dyslexia. *Dev. Sci.* 21:12530. 10.1111/desc.12530 28239921

[B8] Caulfield M. D. McAuley J. D. Servatius R. J. (2013). Facilitated acquisition of eyeblink conditioning in those vulnerable to anxiety disorders. *Front. Hum. Neurosci.* 7:348. 10.3389/fnhum.2013.00348 23847516 PMC3701872

[B9] Cousineau D. Charbonneau D. Jolicoeur P. (2006). Parameterizing the attentional blink effect. *Can. J. Exper. Psychol. Rev. Can. Psychol. Exper.* 60 175–189. 10.1037/Cjep2006017 17076433

[B10] De Quiros G. B. Kinsbourne M. (2001). Adult ADHD: analysis of self-ratings on a behavior questionnaire. *Ann. N. Y. Acad. Sci.* 931 140–147. 10.1111/j.1749-6632.2001.tb05777.x 11462738

[B11] Epstein J. Johnson D. E. Conners C. K. (2001). *Conners’ Adult ADHD Diagnostic Interview for DSM-IV (CAADID).* Toronto: Multi-Health Systems.

[B12] First M. B. Gibbon M. Spitzer R. L. Williams J. B. W. Benjamin L. S. (1997). *Structured Clinical Interview for DSM-IV Axis II Personality Disorders, (SCID-II).* Washington, D.C: American Psychiatric Press Inc.

[B13] First M. B. Spitzer R. L. Gibbon M. Williams J. B. W. (1996). *Structured Clinical Interview For DSM-IV Axis I Disor- ders, Clinician Version (SCID-CV).* Washington, DC: American Psychiatric Press.

[B14] Fostick L. Revah H. (2018). Dyslexia as a multi-deficit disorder: working memory and auditory temporal processing. *Acta Psychol.* 183 19–28. 10.1016/j.actpsy.2017.12.010 29304447

[B15] Germano G. D. Reilhac C. Capellini S. A. Valdois S. (2014). The phonological and visual basis of developmental dyslexia in Brazilian Portuguese reading children. *Front. Psychol.* 5:1169. 10.3389/fpsyg.2014.01169 25352822 PMC4196516

[B16] Gori S. Seitz A. R. Ronconi L. Franceschini S. Facoetti A. (2016). Multiple causal links between magnocellular-dorsal pathway deficit and developmental dyslexia. *Cereb. Cortex* 26 4356–4369. 10.1093/cercor/bhv206 26400914 PMC6317503

[B17] Goswami U. (2015). Sensory theories of developmental dyslexia: three challenges for research. *Nat. Rev. Neurosci.* 16 43–54. 10.1038/nrn3836 25370786

[B18] Green C. S. Bavelier D. (2003). Action video game modifies visual selective attention. *Nature* 423 534–537. 10.1038/nature01647 12774121

[B19] Huettig F. Lachmann T. Reis A. Petersson K. M. (2018). Distinguishing cause from effect - many deficits associated with developmental dyslexia may be a consequence of reduced and suboptimal reading experience. *Lang. Cogn. Neurosci.* 33 333–350. 10.1080/23273798.2017.1348528

[B20] Kibby M. Y. Lee S. E. Dyer S. M. (2014). Reading performance is predicted by more than phonological processing. *Front. Psychol.* 5:960. 10.3389/fpsyg.2014.00960 25285081 PMC4168686

[B21] Kivisaari S. Laasonen M. Leppamaki S. Tani P. Hokkanen L. (2012). Retrospective assessment of ADHD symptoms in childhood: discriminatory validity of finnish translation of the wender Utah rating scale. *J. Attent. Disord.* 16 449–459. 10.1177/1087054710397801 22286113

[B22] Knowlton B. J. Ramus S. J. Squire L. R. (1992). Intact artificial grammar learning in amnesia: dissociation of classification learning and explicit memory for specific instances. *Psychol. Sci.* 3 172–179. 10.1111/j.1467-9280.1992.tb00021.x

[B23] Knowlton B. J. Squire L. R. (1996). Artificial grammar learning depends on implicit acquisition of both abstract and exemplar-specific information. *J. Exper. Psychol. Learn. Mem. Cogn.* 22 169–181. 10.1037/0278-7393.22.1.169 8648284

[B24] Laasonen M. (2002). *Temporal Acuity In Developmental Dyslexia Across The Life Span: Tactile, Auditory, Visual, And Crossmodal Estimations.* Doctoral thesis, University of Helsinki, Helsinki.

[B25] Laasonen M. Hokkanen L. Leppamaki S. Tani P. Erkkila A. T. (2009a). Project DyAdd: fatty acids and cognition in adults with dyslexia, ADHD, or both. *Prostagland. Leukotr. Essent. Fatty Acids* 81 79–88. 10.1016/j.plefa.2009.04.004 19464861

[B26] Laasonen M. Hokkanen L. Leppamaki S. Tani P. Erkkila A. T. (2009b). Project DyAdd: fatty acids in adult dyslexia, ADHD, and their comorbid combination. *Prostagland. Leukotr. Essent. Fatty Acids* 81 89–96. 10.1016/j.plefa.2009.04.005 19523794

[B27] Laasonen M. Leppamaki S. Tani P. Hokkanen L. (2009c). Adult dyslexia and attention deficit disorder in finland-project DyAdd WAIS-III cognitive profiles. *J. Learn. Disabil.* 42 511–527. 10.1177/0022219409345013 19720787

[B28] Laasonen M. Kauppinen J. Leppamaki S. Tani P. Harno H. Hokkanen L. (2012a). Project DyAdd: classical eyeblink conditioning in adults with dyslexia and ADHD. *Exper. Brain Res.* 223 19–32. 10.1007/s00221-012-3237-y 22948736

[B29] Laasonen M. Salomaa J. Cousineau D. Leppamaki S. Tani P. Hokkanen L. (2012b). Project DyAdd: visual attention in adult dyslexia and ADHD. *Brain Cogn.* 80 311–327. 10.1016/j.bandc.2012.08.002 23043869

[B30] Laasonen M. Virsu V. Oinonen S. Sandbacka M. Salakari A. Service E. (2012c). Phonological and sensory short-term memory are correlates and both affected in developmental dyslexia. *Read. Writ.* 25 2247–2273. 10.1007/s11145-011-9356-1

[B31] Laasonen M. Lahti-Nuuttila P. Virsu V. (2002a). Developmentally impaired processing speed decreases more than normally with age. *Neuroreport* 13 1111–1113. 10.1097/00001756-200207020-00008 12151751

[B32] Laasonen M. Service E. Virsu V. (2002b). Crossmodal temporal order and processing acuity in developmentally dyslexic young adults. *Brain Lang.* 80 340–354. 10.1006/brln.2001.2593 11896646

[B33] Laasonen M. Lehtinen M. Leppamaki S. Tani P. Hokkanen L. (2010). Project DyAdd: phonological processing, reading, spelling, and arithmetic in adults with dyslexia or ADHD. *J. Learn. Disabil.* 43 3–14. 10.1177/0022219409335216 19723980

[B34] Laasonen M. Service E. Virsu V. (2001). Temporal order and processing acuity of visual, auditory, and tactile perception in developmentally dyslexic young adults. *Cogn. Affect. Behav. Neurosci.* 1 394–410. 10.3758/Cabn.1.4.394 12467091

[B35] Laasonen M. Vare J. Oksanen-Hennah H. Leppamaki S. Tani P. Harno H. (2014). Project DyAdd: implicit learning in adult dyslexia and ADHD. *Ann. Dyslexia* 64 1–33. 10.1007/s11881-013-0083-y 24162872

[B36] Lazaroo N. K. Bates T. C. Hansell N. K. Wright M. J. Martin N. G. Luciano M. (2019). Genetic structure of IQ, phonemic decoding skill, and academic achievement. *Front. Genet.* 10:195. 10.3389/fgene.2019.00195 30949193 PMC6436069

[B37] Lefly D. L. Pennington B. F. (2000). Reliability and validity of adult reading history questionnaire. *J. Learn. Disabil.* 33 286–296. 10.1177/002221940003300306 15505966

[B38] Lobier M. Valdois S. (2015). Visual attention deficits in developmental dyslexia cannot be ascribed solely to poor reading experience. *Nat. Rev. Neurosci.* 16 225–225. 10.1038/nrn3836-c1 25790867

[B39] Mannel C. Meyer L. Wilcke A. Boltze J. Kirsten H. Friederici A. D. (2015). Working-memory endophenotype and dyslexia-associated genetic variant predict dyslexia phenotype. *Cortex* 71 291–305. 10.1016/j.cortex.2015.06.029 26283516

[B40] Mascheretti S. Gori S. Trezzi V. Ruffino M. Facoetti A. Marino C. (2018). Visual motion and rapid auditory processing are solid endophenotypes of developmental dyslexia. *Genes Brain Behav.* 17 70–81. 10.1111/gbb.12409 28834383

[B41] Moll K. Göbel S. M. Gooch D. Landerl K. Snowling M. J. (2016). Cognitive risk factors for specific learning disorder: processing speed, temporal processing, and working memory. *J. Learn. Disabil.* 49 272–281. 10.1177/0022219414547221 25124507

[B42] Morris R. D. Fletcher J. M. (1988). Classification in neuropsychology: a theoretical framework and research paradigm. *J. Clin. Exp. Neuropsychol.* 10 640–658. 10.1080/01688638808402801 3066798

[B43] Muller B. Wilcke A. Boulesteix A. L. Brauer J. Passarge E. Boltze J. (2016). Improved prediction of complex diseases by common genetic markers: state of the art and further perspectives. *Hum. Genet.* 135 259–272. 10.1007/s00439-016-1636-z 26839113 PMC4759222

[B44] Neef N. E. Muller B. Liebig J. Schaadt G. Grigutsch M. Gunter T. C. (2017). Dyslexia risk gene relates to representation of sound in the auditory brainstem. *Dev. Cogn. Neurosci.* 24 63–71. 10.1016/j.dcn.2017.01.008 28182973 PMC6987796

[B45] Nicolson R. I. Fawcett A. J. (2007). Procedural learning difficulties: reuniting the developmental disorders? *Trends Neurosci.* 30 135–141. 10.1016/j.tins.2007.02.003 17328970

[B46] Nicolson R. I. Fawcett A. J. (2011). Dyslexia, dysgraphia, procedural learning and the cerebellum. *Cortex* 47 117–127. 10.1016/j.cortex.2009.08.016 19818437

[B47] Nicolson R. I. Fawcett A. J. Dean P. (2001). Developmental dyslexia: the cerebellar deficit hypothesis. *Trends Neurosci.* 24 508–511. 10.1016/s0166-2236(00)01896-8 11506881

[B48] Pennington B. F. (2006). From single to multiple deficit models of developmental disorders. *Cognition* 101 385–413. 10.1016/j.cognition.2006.04.008 16844106

[B49] Pennington B. F. Bishop D. V. M. (2009). Relations among speech, language, and reading disorders. *Annu. Rev. Psychol.* 60 283–306. 10.1146/annurev.psych.60.110707.163548 18652545

[B50] Price K. M. Wigg K. G. Feng Y. Blokland K. Wilkinson M. He G. M. (2020). Genome-wide association study of word reading: overlap with risk genes for neurodevelopmental disorders. *Genes Brain Behav.* 19:e126481. 10.1111/gbb.12648 32108986

[B51] R Core Team (2018). *R: A Language and Environment for Statistical Computing.* Vienna, Austria: R Foundation for Statistical Computing. Available online at: http://www.R-project.org/

[B52] Ramus F. Ahissar M. (2012). Developmental dyslexia: the difficulties of interpreting poor performance, and the importance of normal performance. *Cogn. Neuropsychol.* 29 104–122. 10.1080/02643294.2012.677420 22559749

[B53] Ramus F. Szenkovits G. (2008). What phonological deficit? *Q. J. Exp. Psychol.* 61 129–141. 10.1080/17470210701508822 18038344

[B54] Rutter M. Yule W. (1975). The concept of specific reading retardation. *J. Child Psychol. Psychiatry* 16 181–197. 10.1111/j.1469-7610.1975.tb01269.x 1158987

[B55] Sarkio A. (2009). *Voiko Magnosolujen Heikkous Selittää Kehityksellistä Dysleksiaa?.* Master’s thesis, University of Helsinki, Helsinki.

[B56] Scrucca L. Fop M. Murphy T. B. Raftery A. E. (2016). mclust 5: clustering, classification and density estimation using gaussian finite mixture models. *R J.* 8 289–317. 27818791 PMC5096736

[B57] Service E. Laasonen M. (2019). “Luki-vaikeuden tausta eri kielissä ja vaikeudet suomalaisilla lukijoilla. (The basis of dyslexia in different languages and the difficulties in Finnish readers),” in *Luki-Vaikeudesta Luki-Taitoon. (From Dyslexia To Literacy Skills)*, eds Takala M. Kairaluoma L. (Helsinki: Gaudeamus), 81–102.

[B58] Skeide M. A. Kirsten H. Kraft I. Schaadt G. Muller B. Neef N. (2015). Genetic dyslexia risk variant is related to neural connectivity patterns underlying phonological awareness in children. *Neuroimage* 118 414–421. 10.1016/j.neuroimage.2015.06.024 26080313

[B59] Skeide M. A. Kraft I. Muller B. Schaadt G. Neef N. E. Brauer J. (2016). NRSN1 associated grey matter volume of the visual word form area reveals dyslexia before school. *Brain* 139 (Pt 10), 2792–2803. 10.1093/brain/aww153 27343255

[B60] Snowling M. J. (1995). Phonological processing and developmental dyslexia. *J. Res. Read.* 18 132–138. 10.1111/j.1467-9817.1995.tb00079.x

[B61] Snowling M. J. (2008). Specific disorders and broader phenotypes: the case of dyslexia. *Q. J. Exp. Psychol.* 61 142–156. 10.1080/17470210701508830 18038345

[B62] Snowling M. J. Melby-Lervåg M. (2016). Oral language deficits in familial dyslexia: a meta-analysis and review. *Psychol. Bull.* 142 498–545. 10.1037/bul0000037 26727308 PMC4824243

[B63] Stefanac N. Spencer-Smith M. Brosnan M. Vangkilde S. Castles A. Bellgrove M. (2019). Visual processing speed as a marker of immaturity in lexical but not sublexical dyslexia. *Cortex* 120 567–581. 10.1016/j.cortex.2019.08.004 31536945

[B64] Tabachnick B. G. Fidell L. S. (2014). *Using Multivariate Statistics.* Essex: Pearson education limited.

[B65] Tallal P. (1980). Auditory temporal. *Brain Lang.* 9 182–198. 10.1016/0093-934x(80)90139-X 7363063

[B66] Torgesen J. K. Wagner R. K. Rashotte C. A. (1994). Longitudinal-studies of phonological processing and reading. *J. Learn. Disabil.* 27 276–286. 10.1177/002221949402700503 8006506

[B67] Ullman M. T. (2004). Contributions of memory circuits to language: the declarative/procedural model. *Cognition* 92 231–270. 10.1016/j.cognition.2003.10.008 15037131

[B68] Ullman M. T. Pullman M. Y. (2015). A compensatory role for declarative memory in neurodevelopmental disorders. *Neurosci. Biobehav. Rev.* 51 205–222. 10.1016/j.neubiorev.2015.01.008 25597655 PMC4359651

[B69] Valdois S. Bidet-Ildei C. Lassus-Sangosse D. Reilhac C. N’Guyen-Morel M. A. Guinet E. (2011). A visual processing but no phonological disorder in a child with mixed dyslexia. *Cortex* 47 1197–1218. 10.1016/j.cortex.2011.05.011 21704984

[B70] Virsu V. Lahti-Nuuttila P. Laasonen M. (2003). Crossmodal temporal processing acuity impairment aggravates with age in developmental dyslexia. *Neurosci. Lett.* 336 151–154. 10.1016/s0304-3940(02)01253-3 12505615

[B71] Wagner R. K. (1986). Phonological processing abilities and reading: implications for disabled readers. *J. Learn. Disabil.* 19 623–630. 3540167 10.1177/002221948601901009

[B72] Ward M. F. Wender P. H. Reimherr F. W. (1993). The Wender Utah rating scale: an aid in the retrospective diagnosis of childhood attention deficit hyperactivity disorder. *Am. J. Psychiatry* 150 885–890. 10.1176/ajp.150.6.885 8494063

[B73] Wechsler D. (1999). *WASI: Wechsler Abbreviated Scale Of Intelligence.* San Antonio, TX: PEARSON.

[B74] Wechsler D. (2005). *Wechsler Adult Intelligence Scale - Third Edition: Manual.* Helsinki: Psykologien Kustannus Oy.

[B75] Wolf M. (1986). Rapid alternating stimulus naming in the developmental dyslexias. *Brain Lang.* 27 360–379. 10.1016/0093-934x(86)90025-8 3513900

[B76] Woodruff-Pak D. S. (2002). “Human eyeblink classical conditoning in normal aging and Alzheimer’s disease,” in *Eyeblink Classical Conditioning*, eds Woodruff-Pak D. S. Steinmetz J. E. (Boston, MA: Springer).

[B77] World Health Organization (1998). *The International Statistical Classification Of Diseases And Related Health Problems, 10th Revision.* Geneva: World Health Organization.

[B78] Zoubrinetzky R. Collet G. Serniclaes W. Nguyen-Morel M.-A. Valdois S. (2016). Relationships between categorical perception of phonemes, phoneme awareness, and visual attention span in developmental dyslexia. *PLoS One* 11:e0151015. 10.1371/journal.pone.0151015 26950210 PMC4780782

