# Supplementary material for: Project DyAdd: Non-linguistic Theories of Dyslexia Predict Intelligence
Source: Front Hum Neurosci. 2020 Aug 14;14:316. doi: 10.3389/fnhum.2020.00316 (PMC7456923; doi:10.3389/fnhum.2020.00316)
Supplement: Supplementary file 1 [file Table_1.docx]

Appendix 1 Neuropsychological domains used in the analyses together with their sub-components (numbered), individual tasks, and variables (in parentheses)

------------------------------------------------------------------------------------------------------------------------------------------------------------------

Phonological processing, average of (α = .8)

1. Awareness, accuracy (synthesis (correct), Laasonen, 2002; and Pig Latin (correct), Nevala, Kairaluoma, Ahonen, Aro, & Holopainen, 2006)
2. Memory, accuracy (pseudoword span length (correct), Service, Maury, & Luotoniemi, 2007; WMS-III digit span forward length (correct), Wechsler, 2008)
3. Naming, speed (Stroop color naming (speed), Lezak, Howieson, Loring, Hannay, & Fischer, 2004; RAS (speed for two trials), Wolf, 1986)

Technical reading, average of (α = .9)

1. Speed (narrative text (speed), Laasonen, 2002; word list and pseudoword list reading (speed), Nevala et al., 2006)
2. Accuracy (segregating word chains (correct) and searching for misspellings (correct), Holopainen, Kairaluoma, Nevala, Ahonen, & Aro, 2004; narrative text (correct), Laasonen, 2002; word list and pseudoword list reading (correct), Nevala et al., 2006)

Reading comprehension, average of (α = .6)

1. Speed (searching for incorrect words within a story (speed), Holopainen et al., 2004; forced choice task (speed), Nevala et al., 2006)
2. Accuracy (searching for incorrect words within a story (correct), Holopainen et al., 2004; forced choice task (correct), Nevala et al., 2006)

Spelling, accuracy (pseudoword writing (correct), Holopainen et al., 2004) (α not calculated for the single variable)

Arithmetic, accuracy (RMAT (correct), Räsänen, 2004; WAIS-III Arithmetic (correct), Wechsler, 2005) (α = .8)

Executive functions, average of (α = .147)

1. Set shifting (CANTAB Intra-extra dimensional set shifting (stages completed, total errors adjusted), Cambridge Neuropsychological Test Automated Battery, 2004)
2. Inhibition (Color Trails Test (difference score), D'Elia, Satz, Uchiyama, & White, 1996; Stroop (inhibition errors, difference score), Lezak et al., 2004)
3. Planning (CANTAB Stockings of Cambridge (mean initial thinking time 5 moves, problems solved in minimum moves), Cambridge Neuropsychological Test Automated Battery, 2004)

Attention, average of (α = .6)

1. Sustained (Color Trails Test (speed for first trial), D'Elia et al., 1996; Dual task (sustained attention for dots, sustained attention for numbers), Lezak et al., 2004)
2. Divided (Color Trails Test (speed for second trial), D'Elia et al., 1996; Dual task (divided attention for dots, divided attention for numbers), Lezak et al., 2004)

------------------------------------------------------------------------------------------------------------------------------------------------------------------

NB. Cronbach alpha reliabilities were calculated over the variables within each domain.

**References**

Cambridge Neuropsychological Test Automated Battery. (2004). *CANTABeclipse test administration guide*. Cambridge.

D'Elia, L. F., Satz, P., Uchiyama, C. L., & White, T. (1996). *Color Trails Test. Professional Manual*. Odessa, FL: Psychological Assessment Resources.

Holopainen, L., Kairaluoma, L., Nevala, J., Ahonen, T., & Aro, M. (2004). *Lukivaikeuksien seulontamenetelmä nuorille ja aikuisille*. Jyväskylä: Niilo Mäki Instituutti.

Laasonen, M. (2002). *Temporal acuity in developmental dyslexia across the life span: tactile, auditory, visual, and crossmodal estimations.* (Academic dissertation). University of Finland, Helsinki.

Lezak, M. D., Howieson, D. B., Loring, D. W., Hannay, H. J., & Fischer, J. S. (2004). *Neuropsychological Assessment* (4th ed.). New York: Oxford University Press.

Nevala, J., Kairaluoma, L., Ahonen, T., Aro, M., & Holopainen, L. (2006). *Lukemis- ja kirjoittamistaitojen yksilötestistö nuorille ja aikuisille* (Standardization version ed.). Jyväskylä: Niilo Mäki Instituutti.

Räsänen, P. (2004). *RMAT - Laskutaidon testi 9-12 -vuotiaille*. Jyväskylä: Niilo Mäki Instituutti.

Service, E., Maury, S., & Luotoniemi, E. (2007). Individual differences in phonological learning and verbal STM span. *Memory & Cognition, 35*(5), 1122-1135.

Wechsler, D. (2005). *Wechsler Adult Intelligence Scale - Third Edition: Manual*. Helsinki: Psykologien Kustannus Oy.

Wechsler, D. (2008). *WMS-III manual*. Helsinki: Psykologien Kustannus oy.

Wolf, M. (1986). Rapid alternating stimulus naming in the developmental dyslexias. *Brain & Language, 27*(2), 360-379.
